# Prevalence of spondyloarthritis in inflammatory bowel disease according ASAS and ultrassonography and its correlation with plasma calprotectin

**DOI:** 10.1186/s42358-023-00348-6

**Published:** 2024-04-15

**Authors:** Míriam Küster Huber, Valeria Valim, Érica Vieira Serrano, José Alexandre Mendonça, Rafael Burgomeister Lourenço, Thaisa Moraes Ribeiro Espírito Santo, Hilde Nordal, Maria de Fátima Bissoli, Maria Bernadete Renoldi de Oliveira Gavi

**Affiliations:** 1 Program in Public Health of Health Science Center of Federal University of Espírito Santo (PPGSC-UFES), Vitoria, Espírito Santo Brazil; 2 Medicine Department of Federal University of Espírito Santo (UFES), University Hospital of the Federal University of Espírito Santo (Hucam-Ufes/Ebserh), Vitoria, Espírito Santo Brazil; 3 University Hospital of the Federal University of Espírito Santo (Hucam-Ufes/Ebserh), Vitoria, Espírito Santo Brazil; 4 University Hospital of Pontifícia Universidade Católica (PUC), Campinas, Brazil; 5 Gastroenteroly Department, University Hospital of the Federal University of Espírito Santo (Hucam-Ufes/Ebserh), Vitoria, Espírito Santo Brazil; 6 Haukeland Hospital, University of Bergen, Bergen, Norway; 7 Rheumatology Department, University Hospital of the Federal University of Espírito Santo (Hucam-Ufes/Ebserh), Vitoria, Espírito Santo Brazil

**Keywords:** ASAS, Musculoskeletal ultrasonography, Calprotectin, Spondyloarthritis, Inflammatory bowel disease

## Abstract

**Background:**

Enteropathic spondyloarthritis is underdiagnosed and inflammatory biomarkers and ultrasonography (US) could be useful for screening inflammatory bowel disease (IBD) patients. The objective of this study was to evaluate the prevalence of spondyloarthritis (SpA) in IBD patients, according to the Assessment of SpondyloArthritis International Society (ASAS) criteria and the correlation of results of US of entheses and joints with plasma calprotectin levels.

**Methods:**

This was an observational cross-sectional study. Patients from the IBD outpatient clinic of a reference center were evaluated according to ASAS criteria classification, results of US of entheses and joints, and inflammatory biomarker measurements (erythrocyte sedimentation rates, C-reactive protein levels, fecal and plasma calprotectin levels). A *p* value lower than 0.05 was considered significant.

**Results:**

A total of 30.5% of the studied sample (n = 118) of patients with IBD presented at least one inflammatory musculoskeletal manifestation. The overall prevalence of enteropathic SpA was 13.55%, with 10.16% axial SpA and 4.23% peripheral SpA according to the ASAS criteria. A total of 42.1% of patients had an MASEI score greater than 18, 35.2% had synovitis, and 14.7% had tenosynovitis on US, increasing the frequency of diagnosis of enteropathic SpA to 22.8%. Plasma calprotectin levels were similar to those in healthy controls, and correlated only with the fecal calprotectin level (*p* 0.041).

**Conclusions:**

A total of 13.5% of patients met the criteria in accordance with the ASAS criteria for enteropathic SpA, which increased to 22.8% with the addition of US. The prevalence of enthesitis, synovitis and tenosynovitis by US of symptomatic joints and entheses were 42%, 35% and 14.7% respectively. Plasma calprotectin was correlated with fecal calprotectin but not with inflammatory biomarkers or US or ASAS criteria.

## Background

Inflammatory bowel disease (IBD) consists of ulcerative colitis (UC) and Crohn’s disease (CD), which are recurring and remitting chronic diseases. Even though the most frequent symptoms of IBD are related to the gastrointestinal tract, there are a considerable number of extraintestinal manifestations, including articular, ocular, skin, and hepatobiliary manifestations, in addition to other immune-mediated conditions. Depending on the definition, the prevalence of extraintestinal manifestations ranges from 19 to 40% in patients with IBD [1]. Musculoskeletal symptoms are the most common including arthritis, enthesitis and/or spondylitis associated with IBD. In daily practice, spondyloarthritis (SpA) associated with IBD diagnosis is late and underdiagnosed. As a consequence, patients show more disability and low quality of life [2].

SpA is classified according to axial or peripheral (arthritis and enthesitis) manifestation. Sacroiliitis should be confirmed by conventional radiography or magnetic resonance imaging (MRI). SpA diagnosis is generally overlooked or delayed because it can take years between the beginning of inflammatory low back pain and the development of radiographic sacroiliitis [3]. Conventional radiography is the most commonly used imaging method in clinical practice; however, MRI is the standard method because of its high sensitivity and quality and ability to differentiate acute and chronic abnormalities.

On the other hand, peripheral manifestations such as arthritis and enthesitis are not always characterized only by physical examination. Ultrasonography (US) of joints and entheses is a useful, noninvasive, and easily reproducible technique for diagnosing and tracking patients with peripheral SpA [3].

In addition to imaging exams, laboratory tests such as those for measuring the C-reactive protein (CRP) level and erythrocyte sedimentation rate (ESR) are also used for managing patients with IBD and SpA [4]. However, these markers are nonspecific and increased levels can be observed in many viral and bacterial infections, autoimmune diseases, oncological diseases, and other conditions that result in tissue inflammation [5]. In addition, fecal markers are valuable for IBD considering their specificity toward the gastrointestinal tract. Currently, the most commonly used fecal markers are calprotectin and lactoferrin, which are used for IBD diagnosis, evaluation of disease activity, forecasting its occurrence and response to therapy, and reducing costs [6]. Calprotectin can also be measured in plasma and serum [7]. High serum and/or plasma concentrations are observed in different inflammatory conditions [7].

The objectives of the current study were to determine the prevalence of axial and peripheral SpA in patients with IBD and the frequency of ultrasonographic synovitis and enthesitis and to evaluate plasmatic calprotectin as an inflammatory biomarker in patients with SpA associated with IBD.

## Method

This was an observational cross-sectional study. The participants were from the gastroenterology outpatient clinic of the University Hospital of Federal University of Espírito Santo (Hucam-Ufes/Ebserh). The research project was approved by the ethics committee, approval number 49837115.0.0000.5071/2015. The inclusion criteria were as follows: IBD diagnosis according to clinical, endoscopic and histological criteria and according to the Brazilian Association of Ulcerative Colitis and Crohn’s Disease [8]; at least 18 years-old; and consent to participate in the research through an informed consent form. The exclusion criteria were as follows: 1—neoplasic diseases, 2—other autoimmune inflammatory diseases, 3—previous cardiovascular event, 4—heart, kidney, lung and/or liver failure, 5—age under 18 years, 6—presence of acute infection in the last month or chronic infection in the last 6 months. The exclusion criteria for the control group were the same as those for the patient group. The inclusion criteria for the control group were: 1—absence of gastrointestinal symptoms, 2—absence of musculoskeletal symptoms, 3—absence of past or present inflammatory disease or neoplasia, 4—absence of cardiac, renal or pulmonary insufficiency,5—age over 18 years, 6—absence of acute infection in the last month or chronic infection in the last 6 months.

Demographic and clinical characteristics of evaluated patients were as follows: gender, age, race, education, IBD diagnosis time, mean HBI (Harvey-Bradshaw Index), mean SCAII (Simple Clinical Colitis Activity Index), IBD family history, perianal disease, current treatment, extraintestinal inflammatory manifestation, past psoriasis, current psoriasis, past uveitis, current uveitis.

Patients were evaluated by an experienced rheumatologist to investigate musculoskeletal manifestations and Assessment of SpondyloArthritis International Society (ASAS) criteria of classification for axial and peripheral SpA: inflammatory low back pain, peripheral arthritis, enthesitis, dactylitis, psoriasis, uveitis, satisfactory response to nonsteroidal anti-inflammatory drugs (NSAIDs) and family history of SpA [9, 10]. Additionally, the presence of any other musculoskeletal manifestations (e.g., mechanical axial pain, mechanical peripheral pain and diffuse pain) was checked.

**Fig. 1:**
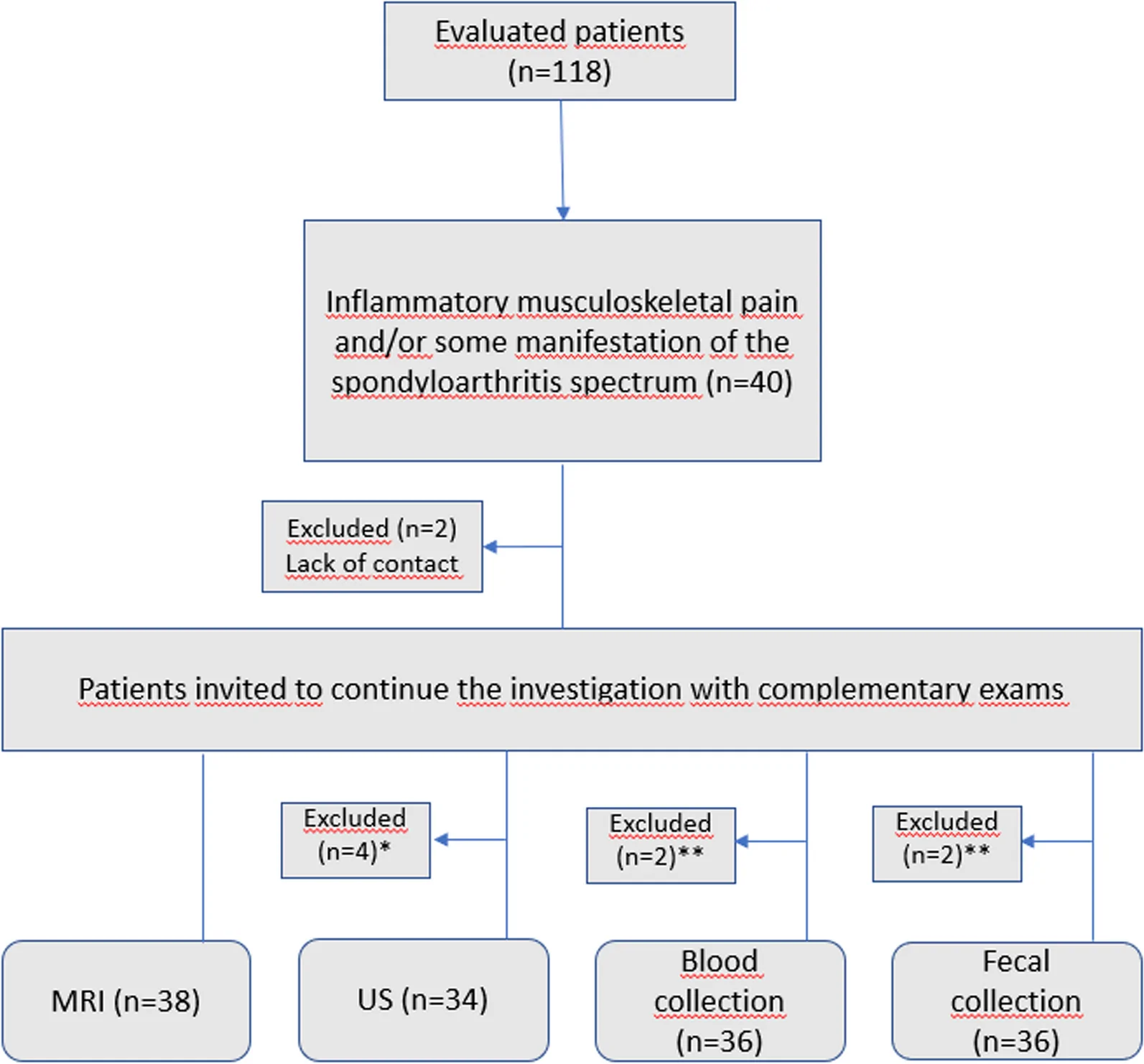
Study design and flowchart. *Excluded (n:4)—2 who lived far from the hospital, 1 had a scheduling conflict and 1 refused to participate. **Excluded (n:2)—2 who lived far from the hospital

Patients who exhibited signals and/or symptoms on the SpA spectrum, such as inflammatory low back pain for more than three months and/or arthralgia, arthritis and/or pain at sites of enthesis evaluated through the Maastricht Ankylosing Spondylitis Enthesitis Score (MASES), and/or enthesitis and/or dactylitis and/or uveitis (present and/or past) were selected for complementary exams (Fig. 1).

The musculoskeletal US protocol was performed by two independent and blinded experienced rheumatologists specializing in US. The machine was the Esaote My Lab 70, linear transducer of frequency L12-18 MHz, power Doppler (PD) of 9–12 MHz and PRF 500–750 MHz, wall filter 1. The clinically symptomatic joints and tendons were evaluated by method B and PD, according to the Outcomes Measures in Rheumatology (OMERACT) definition [11]. Otherwise, the following 12 entheses were explored bilaterally and routinely according to the Madrid Sonographic Enthesis Index (MASEI) score: brachial triceps tendons, distal quadriceps, proximal and distal patellar ligament, distal Achilles tendon, and proximal plantar fascia. The US score evaluated the enthesis thickness, structure, calcifications, erosions, bursae and power Doppler signal. The MASEI score interval was 0 to 136. A cutoff greater than or equal to 18 was used to identify IBD patients with a possible case of SpA [12–14]. Enthesopathy was defined as an abnormal hypoechoic (loss of normal fibrillar architecture) and/or thickened tendon or ligament at its bony attachment (it may occasionally contain hyperechoic foci consistent with calcification), seen in 2 perpendicular planes that may exhibit a Doppler signal and/or bony changes including enthesophytes, erosions, or irregularity, according to OMERACT [9]. Intraclass correlation coefficients (ICCs) were calculated.

Inflammation biomarkers were collected and evaluated at the same time, including C-reative protein (CRP), erythrocyte sedimentation rate (ESR) and fecal and plasma calprotectin. The serum CRP levels were quantified by the latex agglutination test (Wiener lab, Rosário, Argentina) and the ESR was evaluated by the Westergren method. The reference ESR value for men under 50 years old was 15 mm/h, for men between 50 and 85 years old it was 20 mm/h, and for men above 85 years old it was 30 mm/h. The reference for women under 50 years old was 20 mm/h, for women between 50 and 85 years old it was 30 mm/h and for women above 85 years old it was 42 mm/h. The CRP cutoff was lower than 5 mg/L [15].

The plasma calprotectin analysis was performed with ELISA commercial kits (Calprolab, Lysaker, Norway). ELISA was used for quantitative determination of human fecal calprotectin levels (Phadia Laboratory, Portage, Michigan, USA). Plasma calprotectin results were compared with plasma samples from healthy controls matched for sex and age, obtained from a biorepository of the study “Assessment of Cardiovascular Risk in Sjögren's Syndrome”, approved on March 28, 2013, by the ethics committee of the HUCAM, under number 407.199/2013. The cutoff for fecal calprotectin was less then 50mcg/g, and the cutoff for plasma calprotectin was 200 mcg/mL. HLAB27 was obtained through flow cytometry (Fleury, São Paulo, Brasil).

Sacroiliac joint MRI scans of the musculoskeletal system were evaluated by a single experienced radiologist blinded to demographic and clinical information. The definition of "active sacroiliitis on MRI" was bone marrow edema (BMO)/osteitis on a T2-weighted sequence sensitive for free water (such as short tau inversion recovery (STIR) or T2FS) or bone marrow contrast enhancement on a T1-weighted sequence (such as T1FS post-Gd) clearly present and located in a typical anatomical area (subchondral bone) and according to the presence of two lesions on a single coronal slice or a single lesion on two consecutive slices [16, 17].

The descriptive analysis of qualitative variables is shown in tables of frequency and percentages and tables with range, mean, median, and standard deviation for quantitative variables. The *chi*-square test and Fisher’s exact test were used to verify associations among qualitative variables. The Kolmogorov–Smirnov normality test was used to evaluate the normality of the data distribution; considering this outcome, a suitable test to compare groups regarding quantitative variables was chosen. Data that exhibited a normal distribution were tested by Student’s t test to compare their meaning. However, considering the small sample, most tests were nonparametric. In these cases, the Mann‒Whitney test was used to compare two groups. The intraclass correlation coefficient (ICC) was used in the intraobserver reproducibility study of the US analysis at a 10% significance level; therefore, *p* values lower than 0.10 indicated a significant agreement (or disagreement) among the evaluators. A significance level of 5% (*p* values lower than 0.05) was used. All analyses were performed using the statistical software IBM SPSS 20.0 [18].

## Results

We interviewed 118 patients, aged 18–83 years old. Among them, 33.9% (n = 40/118) had inflammatory extraintestinal manifestations associated with SpA, 30.5% (n = 36/118) showed at least one inflammatory musculoskeletal manifestation, 4 patients had uveitis, and 3 patients had psoriasis. We highlight that 17.8% (n = 21/118) had inflammatory low back pain, 15.3% (n = 18/118) had past peripheral arthritis, 2.5% (n = 3/118) had current arthritis, 64.4% (n = 76/118) had articular axial pain and/or peripheral pain and 5.1% (n = 6/118) had diffuse pain. Demographic and clinical characteristics are shown in Tables 1 and 2.

**Table 1:** Demographic and clinical characteristics of evaluated patients (n = 118)

Variables		n	%
Gender	Female	75	63.6
Age (years)	48 ± 15		
Race	Caucasian	56	47.5
	Mixed ethnicity	50	42.4
	Black	12	10.2
Education	Complete primary school	6	5.1
	Incomplete primary school	42	35.6
	Complete high school	38	32.2
	Incomplete high school	12	10.2
	Complete higher education	12	10.2
	Incomplete higher education	8	6.8
IBD	CD	58	49.2
	UC	56	47.5
	Undifferentiated	4	3.4
IBD diagnosis time (years)	10 ± 7.6 (1–36)		
Mean HBI	3.5 ± 2 (1–10)		
Mean SCAII	2.1 ± 1.7 (0–10)		
IBD family history	Yes	17	14.4
Perianal disease	Yes	30	25.4
Current treatment	Topical use	9	7.6
	Sulfasalazine or mesalazine	34	28.8
	Azathioprine	20	16.9
	Isolated anti-TNF	30	25.4
	Azathioprine and sulfasalazine	17	14.4
	NA	8	6.8
Extraintestinal inflammatory manifestation		40	33.9
Past psoriasis		3	2.5
Current psoriasis		0	0
Past uveitis		4	3.4
Current uveitis		0	0

*IBD* Inflammatory Bowel Disease, *CD* Crohn’s Disease, *UC* Ulcerative Colitis, *HBI* Harvey-Bradshaw Index, *SCAII* Simple Clinical Colitis Activity Index, *NA* not available

**Table 2:** Muscleskeletal manifestation in patients with Inflammatory Bowel Disease

Variables		n = 118	%
At least one inflammatory musculoskeletal manifestation		36	30.5
Inflammatory low back pain		21	17.8
Past arthritis		18	15.3
Current arthritis		3	2.5
Past enthesitis		10	8.5
Current enthesitis		2	1.7
Past dactylitis		4	3.4
SpA family history		1	8.0
Satisfactory response to NSAID		10	8.4
Mechanical musculoskeletal pain	Peripheral pain	38	32.2
	Axial pain	23	19.5
	Peripheral and axial pain	15	12.7
	Diffuse pain	6	5.1
	Total	82	69.5
SpA according ASAS		16	13.5
SpA according ASAS + US		29	22.8

*IBD* Inflammatory bowel disease, *NSAIDs* Non-steroidal Anti-inflammatory Drugs, *SpA* spondyloarthritis, *US* Ultrasonography

After clinical evaluation, 33.9% (n = 40/118) of patients with inflammatory musculoskeletal pain and/or some manifestation of the SpA spectrum were invited to continue the investigation with complementary exams (Fig. 1, Tables 3 and 4).

**Table 3:** Demographic and clinical characteristics of patients with IBD and inflammatory manifestations associated to SpA (n = 38)

Variables		n	%
BMI (Grade)	Normal	8	21.1
	Overweight	18	47.4
	Grade 1 obesity	6	15.8
	Grade 2 obesity	2	5.3
	NA	4	10.5
BMI (Mean)	26.73 ± 4.57 (19–37.5)	34	
Gender	Female	27	71.1
Age (years)	45 ± 13 (17–70)		
Race	Caucasian	25	65.8
	Mixed ethnicity	10	26.3
	Black	3	7.9
IBD	CD	24	63.2
	UC	14	36.8
IBD diagnosis time (years)	10.32 ± 7.34 (1–22)		
HBI	4.52 ± 2.21 (1–10)		
SCAII	2 ± 1.12 (0–7)		
Current treatment	Topical	4	10.5
	Sulfasalazine or mesalazine	8	21.1
	Azathioprine	3	7.9
	Anti-TNF	15	39.4
	Azathioprine and sulfasalazine	6	15.8
	NA	2	5.3
At least one inflammatory musculoskeletal manifestation		36	94.7
Previous appointment with rheumatologist		15	39.47
Inflammatory low back pain		19	50.0
Past arthritis		17	44.7
Current arthritis		3	7.9
Past enthesitis		10	26.3
Current enthesitis		2	5.3
Past dactylitis		4	10.5
Past psoriasis		2	5.3
Past uveitis		4	10.5
SpA family history		1	2.6
Mechanical musculoskeletal pain	Peripheral pain	7	18.4
	Axial pain	10	26.3
	Peripheral and axial pain	4	10.5
	Diffuse pain	1	2.6
	Total	22	57.8

*BMI* Body Mass Index, *IBD* Inflammatory Bowel Disease, *CD* Crohn’s disease, *UC* Ulcerative Iolitis, *HBI* Harvey-Bradshaw index, *SCAII* Simple Clinical Colitis Activity Index, *SpA* spondyloarthritis, *NA* Not Available

**Table 4:** Frequencies of HLA-B27 (n = 36) and abnormal MRI for sacroiliitis of patients with IBD (n = 38)

Variables		n	%
HLA-B27	Positive	3	8.3
MRI	Lack of sacroiliitis signs	22	57.9
	Presence of sacroiliitis	10	26.3
	Chronic possible past sacroiliitis	6	15.8

*HLA-B27* Histocompatibility leukocyte antigen B27, *MRI* Magnetic resonance imaging, *IBD* Inflammatory bowel disease

The MASEI scores for entheses and symptomatic joints were investigated by US in 34 patients and showed a mean of 17.79 ± 11.11. Of those, 47% (n = 16/34) of patients showed MASEI scores higher than 18. We highlight that 100% of patients with MASEI scores had at least one case of enthesitis. Among the 408 evaluated entheses, 280 (68%) were abnormal. US of the symptomatic joints was also performed on 22 patients who had arthralgia and showed synovitis in 35.2% (n = 12/34) (15 positive joints on mode B and 1 positive for power Doppler) and in 14.7% (n = 5/34) of patients with tenosynovitis. Likewise, 41.1% (n = 14/34) of patients were using anti-TNF biologic therapy and 55.8% (n = 19/34) of patients were using synthetic drugs (sulfasalazine or methotrexate). Intraobserver agreement was statistically significant (*p* < 0.1) for synovitis, tenosynovitis and MASEI (Table 5).

**Table 5:** Intra-observer agreement for synovitis, tenosynovitis and MASEI (n = 10)

Variables	ICC	IC 95%		*p* value
		Minimum	Maximum	
Synovitis	0.795	0.143	0.950	0.004
Tenosynovitis	0.609	− 0.325	0.898	0.074
MASEI	0.741	− 0.211	0.942	0.002

After clinical and complementary evaluation, 13.5% (n = 16/118) had SpA according to the ASAS criteria, 12 patients received an axial SpA diagnosis, and 5 patients received a peripheral SpA diagnosis (one patient had both).

After US of the entheses and joints, 11 negative cases according to the ASAS criteria, had typical abnormalities. Consequently, 22.8% (n = 29/118) of the total sample had a diagnosis of enteropathic SpA according to a combination of the ASAS criteria and US analysis of symptomatic joints and entheses. Overall, US added 61% more sensitivity to the ASAS classification criteria (*p* ≤ 0.05).

The inflammatory biomarker results are presented in Table 6. The ESR was high in 44.4% (n = 16/36) and CRP in 36.1% (n = 13/36) of patients. We highlight that 69% (n = 24/36) of patients had fecal calprotectin levels higher than 50 mcg/g and 31% (n = 12/36) had fecal calprotectin levels higher than 200 mcg/g.

**Table 6:** Inflammatory biomarkers IBD with SpA clinical manifestations (n = 36)

Variables	Minimum	Maximum	Mean	Median	Standard deviation
ESR*	1.00	96.00	25.22	20.00	21.87
CRP**	0.17	26.75	4.91	3.37	5.46
Fecal calprotectin***	4.00	4931.00	624.95	84.00	1309.40
Plasma calprotectin	216.62	7610.06	786.92	526.28	1214.33

*IBD* Inflammatory Bowel Disease, *SpA* Spondyloarthritis

*ESR increased when above reference value (RV); RV of ESR for men under 50 years until 15 mm/h, 50–85 years until 20 mm/h and above 85 years until 30 mm/h; RV of ESR for women under 50 years until 20 mm/h, 50–85 years until 30 mm/h and above 85 years until 42 mm/h

**CRP increased when above reference value (RV); RV of CRP lower than 5 mg/L

***RV of fecal calprotectin inferior to 50mcg/

Plasma calprotectin levels in patients with IBD with extraintestinal manifestations were similar to those in controls (798 ± 1,182 vs. 662 ± 408, *p* = 0.858). The intestinal disease in the majority of these patients was under control based on the index of intestinal activity (mean HBI of 4.52 and mean SCAII of 2), and 41.7% (n = 15/36) of patients were using anti-TNF therapy.

We found that the correlation between plasma and fecal calprotectin was statistically significant with a *p* value of 0.041, but no correlation was found with other inflammatory biomarkers, including CRP, ESR, or imaging (US or MRI) features (Table 7). Additionally, we did not find a correlation with the ASAS criteria for SpA. However, average plasma calprotectin levels had a superior inclination toward peripheral SpA in comparison with the axial SpA group (1838 vs. 696, *p* = 0.567) (Table 8).

**Table 7:** Correlation between plasma calprotectin and imaging features (US* and MRI**)

Variables		n	Mean	Median	Standard deviation	*p* value
US enthesitis	Negative	17	50.41	25.86	86.66	0.746
	Positive	16	27.90	24.73	17.02	
US synovitis Mode B	Negative	10	29.21	20.48	22.58	0.292
	Positive	13	31.78	26.54	15.81	
US tenosynovitis Mode B	Negative	17	32.54	26.09	19.60	0.327
	Positive	6	25.36	19.51	15.83	
US tenosynovitis Power Doppler	Negative	22	31.37	25.98	18.74	–
	Positive	1	15.14	15.14	–	
	Negative for sacroiliitis	20	47.06	26.40	79.93	
MRI	Positive for sacroiliitis	10	31.47	26.31	20.60	0.842
	Chronic sacroiliitis	6	26.77	23.78	14.56	

*Mann–Whitney test

**Kruskall-Wallis test

**Table 8:** Plasma calprotectin in SpA (n = 36) compared to healthy controls (n = 36)

Groups	Mean	Median	Standard deviation	*p* value
Healthy controls	662.03	499.21	408.51	0.858
SpA	798.08	531.12	1182.03	
Axial SpA	696.11	540.86	438.50	0.703
Peripheral SpA	1838.66	435.20	3230.05	0.498

## Discussion

In clinical practice, extraintestinal manifestations are challenging conditions that require a multidisciplinary approach and specific tools for diagnosis and follow-up to provide the best patient care. Regardless, screening protocols for extraintestinal manifestations and side effects of treatment are still not standardized in clinical trials. The most adequate approach is to refer patients to an experienced specialist in managing these manifestations [19]. In the present work, after a first evaluation of IBD patients, 30.5% exhibited at least one inflammatory musculoskeletal manifestation. This result is similar to other reports in the literature [2, 20–22]. When we analyzed the musculoskeletal complaints of these patients, through anamnesis and clinical exams, 17.8% had inflammatory low back pain, 15.3% had past peripheral arthritis, 2.5% had current arthritis, 26.3% reported a suggestive case of past enthesitis, and 5.3% had current enthesitis. Peripheral arthritis in patients with CD and UC occurs at a frequency that varies between 2.8 and 30% according to many published studies [21, 23–25]. Previous investigations demonstrated that the prevalence of inflammatory low back pain on patients with IBD varies between 9 and 30% [26].

Most of the patients who had SpA were women, mostly with CD. Similar to previous studies, SpA characteristics were more frequently reported in female patients with IBD [2].

We found that the prevalence of enteropathic SpA was 13.55%, whereas 10.16% was axial SpA and 4.23% was peripheral SpA, according to the ASAS criteria. We highlight that one patient fulfilled the criteria for both axial and peripheral SpA. The Norwegian IBSEN study followed IBD patients for 20 years and observed a cumulative prevalence of 27.9% of patients with peripheral SpA, 7.7% of patients with axial SpA, and 11.5% of patients with inflammatory low back pain [27].

Throughout the analysis of patients who received a diagnosis of axial SpA, we found that 10 (26.3%) patients had active sacroiliitis on MRI, and most (n = 9) were not using anti-TNF therapy. This suggests that the prevalence is underdiagnosed because the treatment of intestinal manifestations can reduce musculoskeletal manifestations [28, 29].

The prevalence of HLA-B27 was low (7.9%), but similar to that in another study performed in Brazil, that found 5,6% in the IBD cohort [21]. In contrast, HLA B27 were positive in 29% of the IBD patients in a cohort from the outpatients clinic of Ankara University, Turkey [30].

Karreman et al. [23], in a systematic review and meta-analysis, investigated the prevalence and incidence of SpA in patients with IBD. A total of 71 studies were included, and the grouped prevalence of sacroiliitis was 10% (confidence interval of 95% [CI] 8–12%). Geographic area, definition and use of different criteria contributed to the considerable heterogeneity of the results. This same review observed that there were few estimates for enthesitis, with prevalence varying from 1 to 54%. Enthesopathy and enthesitis can have a clinical diagnosis, but US is a highly sensitive and noninvasive tool that can improve the diagnosis. The prevalence of enthesitis in patients with IBD varies from 5 to 10% and is predominant in those with CD [26]. Bandinelli et al. [31] stated that enthesitis is a specific and sometimes isolated signal from IBD associated with SpA, which is constantly underdiagnosed or mistaken for mechanical chronic pain. In fact, in this study, anamnesis with physical exam identified only 1.7% of patients with enthesitis. Therefore, we highlight the importance of US confirming clinically symptomatic and subclinical enthesitis. After US analysis, 42.1% of patients had an MASEI score of ≥ 18, 35.2% of patients had synovitis, and 14.7% had tenosynovitis. Atzeni et al. [3] found that 33.3% of patients with IBD showed an MASEI score of ≥ 18, suggesting SpA. This information aided our differential diagnosis among possible articular and periarticular inflammatory or mechanical etiologies. Considering that the average age of the study population was 45 years old and that 69.5% were overweight or obese, there was an overlap of inflammatory and mechanical pain etiologies. Considering that noninflammatory arthralgia can be clinically diagnosed and might cause disability in patients with IBD, our study identified that, of the 118 interviewed patients, 64.4% complained of axial and/or peripheral mechanical pain and 5.1% complained of diffuse pain. Most investigations about arthropathy in IBD exclude the noninflammatory causes of articular pain [26]. A hospital-based study (ORCHARD) with 1459 patients with IBD and a population-based study (PALM) with 521 patients with IBD described prevalences of 8% and 16%, respectively, of patients clinically diagnosed with mechanical arthralgia.

Hence, adding US to the ASAS classification criteria increased the prevalence from 13 to 23% of the analyzed sample who received an enteropathic SpA diagnosis. Musculoskeletal ultrasonography can represent a valid complementary and easily available imaging technique to support clinical evaluation in the outpatient setting according to many published studies [32–36].

Surprisingly, 21 of 36 patients (58.3%) who reported some inflammatory musculoskeletal manifestation never consulted a rheumatologist. According to Stolwijk [2], this also occurred in almost 50% of patients who reported musculoskeletal disorders. On the other hand, according to Guillo et al. [1], the importance of rheumatologists’ opinions on investigations with patients with IBD and extraintestinal manifestations is clear. There might be many reasons for this. First, gastroenterologists do not always ask patients with IBD about musculoskeletal manifestations related to SpA or do not know exactly which symptoms belong to the spectrum of autoimmune rheumatic diseases. In addition, symptoms related to SpA have a fluctuating characteristic, and for SpA diagnosis, it is not necessary to have all symptoms at the time of diagnosis. Last, a high percentage of patients receive immunosuppressive therapy, including biological therapy, which may also influence SpAs symptoms; therefore, gastroenterologists may think that referral to a rheumatologist would not change the conduct for these patients [2]. Even then, current norms about extraintestinal manifestations of IBD do not include treatment algorithms to aid professionals in the decision-making process [37]. In this case, it is even more important to refer patients with musculoskeletal manifestations to a rheumatologist for joint management.

Finally, considering that there are numerous heterogeneous studies [7, 37–40] on plasma calprotectin in SpA, and that some investigations include all subtypes of SpA and others are centered only on ankylosing spondylitis, the present work evaluated the characteristics of plasma calprotectin in samples of patients with IBD and articular symptoms. There was no significant statistical correlation among values of plasma calprotectin and inflammatory biomarkers (ESR and CRP) and there was no difference between plasma calprotectin of the studied sample and healthy controls matched by age and sex. Most likely, they included individuals mostly with intestinal and extraintestinal manifestations under control. Future studies should evaluate individuals with moderate to high disease activity. On the other hand, the correlation between plasma and fecal calprotectin was statistically significant with a p value of 0.041. Even though it did not reach significance, it seems that the average plasma calprotectin level is higher in patients with peripheral articular disease. A larger sample size can be used to explore this issue. The literature shows heterogeneous data regarding plasma calprotectin levels (7, 28–31). Some authors report an increase in plasmatic levels of calprotectin in patients with SpA, whereas others have found similar levels between SpA patients and controls [7]. Other investigations have reported lower or similar levels in patients with SpA in comparison to patients with rheumatoid arthritis [7]. Cypers et al. [38] found increased plasmatic levels of calprotectin associated with peripheral involvement in SpA, which could explain the near normality of the observed levels in patients with only axial involvement. However, this observation was not confirmed by De Rycke et al. [40], and more data are needed. Moreover, in another study, plasma calprotectin was normal in ankylosing spondylitis, in contrast with various other inflammatory rheumatic diseases [39]. It was already demonstrated that plasma calprotectin is positively related to CRP, ESR, leukogram and platelets in patients with SpA. Nonetheless, it does not seem to be a reliable biomarker of SpA disease activity, considering that almost no correlation has been found between plasma calprotectin and the BASDAI score of disease activity and BASFI function score [7]. Ometto et al. [7] still reported that plasma calprotectin decreases rapidly after efficient treatment with TNF inhibitors, and in our samples, 39.47% of patients were under therapy with anti-TNF, which could explain the lack of correlation between them and the control group. According to Kalla et al. [41], plasma calprotectin was correlated with current biomarkers such as CRP and fecal calprotectin. In our study, plasma calprotectin showed a significant statistical correlation with fecal calprotectin but did not have a significant correlation with other biomarkers.

Another limitation is the fact that the analyzed sample was a convenience sample; therefore, patients who agreed to participate in the research could be those with a greater chance of having an articular complaint, increasing the frequency of observing musculoskeletal manifestations. It is necessary to consider that the specialist opinion was clearly influenced by the results of articular and enthesis US.

Last, the sensitivity and specificity of the ASAS classification criteria for axial SpA were 83% and 84%, respectively, and the imaging arm has been further scrutinized because evaluation of patients with nonspecific back pain has demonstrated a positive MRI in 20% of patients [42].

Finally, subjective measures and patient-reported outcomes may overestimate disease activity, and thus are unreliable in therapeutic decision making in clinical practice, since they may lead to intensification of, or switching, immunotherapy when it is not necessarily warranted. Clinical data complemented by more objective measures of inflammation such as entheseal sonography can help clarify the diagnosis [35].

## Conclusions

One third of patients with IBD exhibited inflammatory musculoskeletal manifestations suggesting SpA, and 13.5% met the ASAS criteria for enteropathic SpA, wich increased to 22.8% when US results were added. The prevalence of enthesitis, synovitis and tenosynovitis (14.7%) by US of symptomatic joints and entheses were 42%, 35% and 14.7% respectively. Plasma calprotectin was correlated with fecal calprotectin but not with inflammatory biomarkers or US or ASAS criteria.

A good history and detailed physical examination can alarm clinicians about musculoskeletal symptoms in patients with IBD. This generates an opportunity to guarantee an adequate and early referral to the rheumatology service from the gastroenterology department, as well as potential therapeutic adjustments and physical habits and lifestyles. These results clearly demonstrate the usefulness of Ultrasonography (US) of joints and entheses is noninvasive, and easily reproducible technique for diagnosing and tracking patients with peripheral SpA.

## Data Availability

The data that support the findings of this study are available on request from the corresponding author.

## Abbreviations

SpA: Spondyloarthritis
IBD: Inflammatory bowel disease
MRI: Magnetic resonance Imaging
US: Ultrasound
CRP: C-reactive protein
ESR: Erythrocyte sedimentation
ASAS: Assessment of SpondyloArthritis international Society
HUCAM: Hospital Universitário Cassiano Antônio de Moraes
NSAIDs: Non-steroidal anti-inflammatory drugs
MASEI: Madrid sonographic enthesis index
PD: Power doppler
ICC: Interclass correlation coefficient
CD: Crohn’s disease
UC: Ulcerative colitis
HBI: Harvey-Bradshaw index
SCAII: Simple clinical colitis activity index
BMI: Body mass index
OMERACT: Outcomes measures in rheumatology
